# Book review of *Structural Bioinformatics: An Algorithmic Approach *by Forbes J. Burkowski

**DOI:** 10.1186/1475-925X-8-14

**Published:** 2009-07-24

**Authors:** Ilza K Pajeva

**Affiliations:** 1 Centre of Biomedical Engineering, Bulgarian Academy of Sciences, 1113 Sofia, Bulgaria

The field of structural bioinformatics has been extensively explored in the recent years. A glance into the on-line book store Amazon.com, Inc. shows several hundred titles related to this topic. Ordering them according to the relevance gives the Burkowski's book the 2^nd ^position. To make a profound analysis of the book and to compare it to the others published so far suggests that the reviewer should have been read many of them. Unfortunately, this is not the case. Therefore, I can give an opinion based on my own experience as a person working in a related field and from reading some freely available selected parts of other related books.

The first difference between this and other books on the same topic comes from the fact that it is written by a person who is a professional in mathematical modeling and computational algorithms. The author intentionally added "An algorithmic approach" to the title to outline the fact that, in contrast to other books on structural bioinformatics, the accent will be given to key algorithms used for solving problems related to macromolecuar structures. The other difference is that he is a single author and this gives him advantage to design the book according to his personal preferences and understanding of the topic. These two facts feature the book and make it unique.

The book is suitable for everyone who would like to make some progress in studying structural biology by experiencing a different approach to its problems. In any case it stimulates the analytical thinking of those who read. The reading could be addressed to wide auditorium and this comes from the interdisciplinary nature of structural bioinformatics that demands knowledge in mathematics, biology, chemistry, informatics. The book provides a good framework of an interdisciplinary course in the field, thus it is suitable for undergraduate students, but also for master and PhD students. Professionals from the industry and academic researchers could be potential readers too.

The book consists of 10 chapters and most of them have an introduction called "motivation" and exercises in the end. The motivation, as the name itself suggests, gives reasons to define the problem addressed in the chapter, as belonging to the algorithmic structural bioinformatics. To be answered the questions and tasks in the exercises require careful reading and active thinking. Some of them could be a challenge even for more experienced readers.

In the chapters, when possible, the author tries to consider the problem from the point of view of three main realms: *Nature *(the source of empirical observations), *Science *(the mathematical modeling of the natural process), and *Computation *(the science of calculating predictions and mathematical objects based on mathematical models). These three terms are generally outlined in the 1^st ^chapter. The three levels of scientific methodology to study structural bioinformatics are illustrated by a detailed analysis of force fields. Some book readers, and especially those of the younger generations, could be pleased by the analogy made to famous movies and examples from the literature when explaining the modeling issues: the "Rashomon effect" or how can we know when the explanation is true (reliability); "Ockham's razor" or how to select the model of highest simplicity while still maintaining the power of the model to represent the observables seen in the natural process (simplicity); and the Bellman's "curse of dimensionality" or the dangers of using overcomplicated models (dimensionality). Other points of modeling like interpretability, refutability, complex and approximation, sources of errors are also addressed in this chapter.

Chapter 2 is an introduction to the molecular substructure. Such description can be found in many other books and in more details. Here, however, the presentation is somewhat different from the standard lessons on molecular structures – the exceptions rather than the rules are discussed, e.g. in the classification of the amino acids, the description of the alpha – helix etc. On the example of Pauling's discovery of the alpha-helix the author shows how thinking in a nonstandard may lead to discovery. The "problematic" design of proteins (presence of redundant substructures) is explained by their "function-driven" evolution. Some questions of protein functionality are also discussed, although, as the author notices, the book does not consider functionality in any significant depth. Besides the primary, secondary, tertiary, and quaternary protein structure, the protein domain is also considered as another structural concept important for understanding the protein functionality. Next, an overview of the RNA structure is given and its role for the catalytic capabilities of the acid is discussed.

Logically, the next chapter deals with the data sources, formats and applications. Such knowledge is a necessary step for all working in the area of structural bioinformatics to realize algorithms important for: (i) manipulating structural data; (ii) detecting structural patterns in the macromolecules; (iii) understanding the cellular processes. The chapter focuses on structure databases. PDB (Protein Data Bank) is given a special attention as a source of structural data. A citation from the RCSB website outlines the important role of this source for the modern research and education: over 10,000 scientists, students, and educators visit the PDB web site every day, and, on average, 2.2 files are downloaded every second (see the footnote on p. 84). The PDB summary (PDBsum) web site is recommended as an excellent starting point for navigation in the information ocean of the proteins. Next, SCOP, CATH, PubChem and DrugBank are shortly described. Free available visualization software packages are listed and several aspects are discussed when comparing different programs available: plug-in versus stand-alone; viewing perspective, graphical presentation, visual effects and computational tools. Additionally, there is a list of software packages for structural bioinformatics that are free available including also open source software (PyMOL, Eclipse, Marvin Sketch, ACD/ChemSketch, JOELib2, CDK, BioPython).

Chapter 4 is the heart of the book. It describes the algorithm used for solving optimization problems known as "dynamic programming" (DP) or, in other words, "multistage decision processes". DP is considered as the most useful paradigm for designing algorithms by bioinformaticians. The main idea of DP is explained on an example out of a bioinformatics content but good enough for illustration of the main ideas of DP. The two main aspects of the optimization problem (configurations and scores) and the problem analysis are described on a hypothetical Al Gore rhythm for giving talks against global warming. This example does not make structural bioinformatics equal to politics but do suggest that both could be optimized. An application of the DP methodology, however, is done for solving of a problem with bioinformatics content: measurement of similarity between two nucleotide sequences – the longest common subsequence problem.

The prediction of the secondary structure of RNA (Chapter 5) is the first example to fully illustrate the author's approach to algorithmizational structural bioinformatics. Here the author goes back to the main realms (*Nature, Science and Computation*) introduced in Chapter 1. *Nature *is related to hydrogen bond formation, energy issues (in a thermodynamic sense) and consensus sequence patterns; *Science *relates to thermodynamically based modeling of the secondary structure; *Computations *deal with the energy minimization strategy. The dot plots suitable for mathematical analysis are shown as alternate representations of the modeled secondary structure. The main focus of the chapter is the DP approach to the Nussinov's and MFOLD algorithms for predicting secondary structure.

The next DP application is for solving the problem of protein sequence alignment (Chapter 6). This task is important for those who do homology modeling and it is driven by the fact that the 3D structures of two proteins diverge much more slowly in the evolutionary process than does the amino acid sequence identity between the proteins. Here again the problem is considered in the three realms. In *Nature *the problem can be described by giving binary values when answering the question about whether the proteins are homologous (value 1) or not (value 0) (there is no exact solution of this problem, because it is complex in its core). Presented in this way the problem can be mathematically formulated in *Science *as attending maximal similarity between the compared sequences by introducing gaps and penalizing these gaps. *Computation *describes the optimal global alignment problem in terms of DP.

Chapter 7 introduces the modeling of static protein structures with known 3D positions of the protein atoms: computations of bond lengths and interatomic distances, bond angles and dihedral angles. The Ramahandra plot (generated by PROCHECK) as a tool for validation of the predicted protein geometry and the inertial axes for approximation of the secondary structural elements of the protein are also discussed.

The next two chapters deal with the mathematics of coordinate transformation (Chapter 8) and its applications for structural alignment (Chapter 9). Translation, rotation and isometric transformations are described in details suggesting a basic knowledge of linear algebra. As noticed by the author, the selected applications are chosen by his preference. Nevertheless, the exposé gives the most important knowledge in the structural comparison: techniques, scoring similarities, superposition algorithms (with a detailed description of their formalism) and algorithms comparing relationships within proteins, namely Dali (Distance alignment) and SSAP (Secondary Structure Alignment Program) algorithms.

The book ends with introduction to machine learning. The computational issues of linear regression, ridge regression and kernel functions are presented. An implementation of the SVM (Support Vector Machine) classification algorithm, that became very popular in bioinformatics over the last few years, is described using a kernel methods approach.

The Appendices supply the reader with the basics of linear algebra and are for readers familiar with matrix algebra, vector analysis, linear transformations, and related mathematics.

The book gives a new point of view to the widely explored field of structural bioinformatics. It is worth noting that writing books on this strongly interdisciplinary topic is a challenge for any author (and also for those who review them). The book of Burkowski has its unique place in structural bioinformatics field. I recommend this book for reading, and, I believe, everyone who does will find his own way of making use of it.

